# Female Physicians

**Published:** 1867-06

**Authors:** 


FEMALE PHYSICIANS.

Dr. Atlee called from the table his preamble and resolutions, proposing to recognize female physicians by the same rules and limitations as other physicians. The vote on taking them from the table was 57 ayes to 52 nays.

As there was no time for satisfactory discussion, Dr. Atlee moved the previous question, but it was not sustained.

Dr. Pallen, of St. Louis, said:—"At home, I am considered a friend of the ladies, and nothing would give me greater pleasure than to advocate their claims, where it could be done legitimately. But in Europe, Austria, France, and Prussia, the practice of medicine and obstetrics by females has proved a total failure. It is contrary to the spirit of our profession to have anybody connected with it except men, and men of reason. Nature has so ordained the female that, at certain periods, she is absolutely unfitted to do anything, consequently, could not attend to the legitimate duties of the physician. Another serious objection—no person can practiee medicine or surgery without a knowledge of pathological anatomy, and no woman having sufficient delicacy to enter the sick chamber, would enter the dissecting room to obtain such knowledge. Imagine a lady, with her style of dress, flitting around in the charnel house, or with microscopes diving into cancer cells. Pass such a resolution as this, and a thousand women about the country, practicing specialties connected with the female organization, will demand recognition at our hands, and claim authority for their business pretensions. I think the resolution should be voted down, because it is contrary to the laws of nature for women to practice medicine.

Dr. Davis stated that at this late period of the session there was not time to enter upon any general discussion of the subject before them, and, consequently, he moved to refer the preamble and resolution offered by Dr. Atlee to the Committee on Ethics.

Dr. Bowditch advocated the adoption of the resolution.

But the motion to refer to the Committee on Ethics was carried by a large majority.

